# The Value of Teledermatology Advice for Skin Toxicity in Oncology: Experience From a Pilot Study

**DOI:** 10.2196/40053

**Published:** 2024-02-29

**Authors:** Sofie Mylle, Jorien Papeleu, Isabelle Hoorens, Evelien Verhaeghe, Lieve Brochez

**Affiliations:** 1 Dermatology Department Ghent University Hospital Ghent Belgium; 2 Cancer Research Institute Ghent Ghent Belgium

**Keywords:** e-health, teledermatology, oncology, epidermal growth factor receptor, EGFR-inhibitors, skin toxicity, cancer, dermatology, therapy, pilot study

## Introduction

Epidermal growth factor receptor (EGFR) inhibitors are increasingly used in oncologic treatments. Skin toxicity is a possible side effect and can seriously impair quality of life (QoL) and result in treatment tapering or discontinuation [1-4]. Despite several preventive and treatment guidelines, oncologists encounter difficulties in managing skin toxicities [5,6]. In Belgium, this struggle is compounded by some hospitals having no or only part-time in-house dermatologists. We initiated a teledermatology pilot project in 3 Belgian hospitals with no or limited access to dermatological advice and evaluated its value in anti-EGFR–induced skin toxicity for both patients and oncologists.

## Methods

### Overview

Patients receiving anti-EGFR treatment and developing skin toxicity were eligible. Clinical imaging data were exchanged through an existing secured platform (Mediris). Three oncologists from 3 different Belgian nonuniversity hospitals participated. Clinical information and images were uploaded to the platform and sent to the teledermatologists. Three dermatologists from Ghent University Hospital were involved as teledermatologists and formulated their advice within 48 hours. Questionnaires on expectations and satisfaction with the teledermatology platform were completed by both patients and oncologists at the start and end of the study.

### Ethical Considerations

Ethical approval was obtained from Ghent University Hospital (EC2018/0984) and participating hospitals, and participants provided written informed consent.

## Results

The study started in January 2019 and was prematurely terminated in mid-March 2020 because of the COVID-19 pandemic. In total, 35 store-and-forward consultations were performed for 6 patients. The most frequent reasons for advice involved xerosis or eczema (n=27, 77%) and papulopustular rash (n=18, 51%). All patients had grade 2 toxicity according to the CTCAE (Common Terminology Criteria for Adverse Events; version 5.0).

Three out of 6 patients completed the questionnaires; they were overall positive about the project and felt that teledermatology was reliable, valuable, and efficient. Although all the participating oncologists reported difficulties in accessing dermatological advice, they used the teledermatology platform less than anticipated. They all reported uploading of images and patient information to be difficult and time-consuming. Nevertheless, the oncologists noted that teledermatology was as valuable (1/3) or more valuable (2/3) than expected.

In 37% (13/35) of all teleconsultations, teledermatologists reported that more information was needed to provide tailored advice. In 29% (10/35) of consultations, teledermatologists indicated that a live consultation would have been relevant, either to collect additional information for decision-making or to explain and motivate the patient about a specific treatment.

## Discussion

Although skin toxicity during anti-EGFR treatment might be considered a minor, non–life-threatening side effect, it is known to markedly impact patients’ QoL. This may lead to dose tapering or early treatment discontinuation, thereby potentially interfering with its anticancer effects [1-4]. Skin toxicity is reported as being more discouraging than complete hair loss and as discouraging as nausea [6]. Oncologists intend to initiate skin-focused treatment in cases of skin toxicity of grades 2 and 3 and only refer 8% of their patients for specialized dermatological advice [4]. This small multicenter pilot study aimed to investigate the value of teledermatology to facilitate dissemination of dermatological advice to patients treated with EGFR inhibitors.

**Figure 1:**
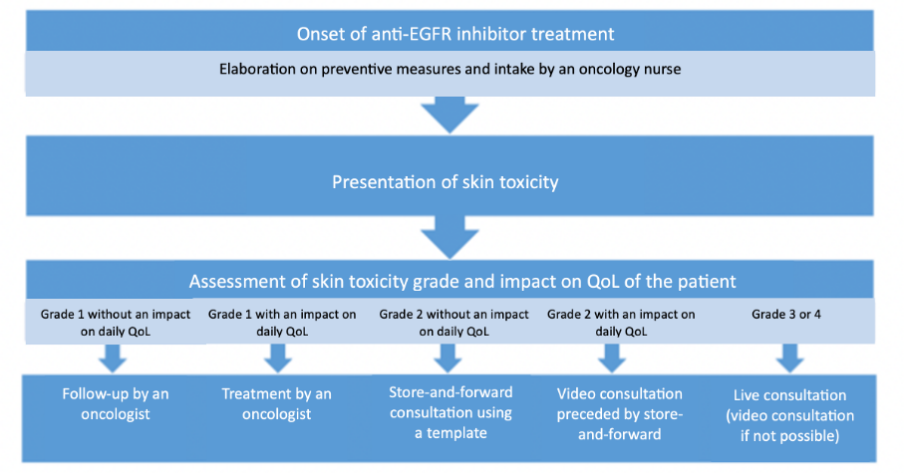
Proposition of the ideal workflow for the management of anti-EGFR–related skin toxicity. EGFR: epidermal growth factor receptor; QoL: quality of life.

From January 2019 until mid-March 2020, overall 35 teleconsultations were provided to 6 patients. Images and clinical information were uploaded to a secured eHealth platform and evaluated by a teledermatologist within 48 hours. Unfortunately, the enrollment was lower than anticipated, most probably because the teledermatology platform was perceived as non–user-friendly. The teledermatologists reported clinical information to be missing in about one-third of the teleconsultations. They indicated the lack of direct communication to promote diagnostic accuracy and therapeutic adherence. A suggested workflow is depicted in Figure 1. Store-and-forward teledermatology has been shown to be able to improve the efficiency of and access to care [7]. The COVID-19 lockdown has demonstrated that teledermatology can help in minimizing unnecessary in-person visits. Many skin conditions may be adequately managed remotely, while others may be selected for an additional step (triaging). This could imply a physical or video consultation to advise patients in other hospitals or at home.

Although several guidelines on skin toxicity management are available, skin toxicity and its impact on QoL seem not always properly recognized. Teledermatology may offer benefits including reduced waiting times, travel costs and sanitary costs, and equalization of access to specialist advice. In this pilot study, both oncologists and patients acknowledged the added value of teledermatological advice on skin toxicity during anti-EGFR therapy. However, several shortcomings of a store-and-forward consultation are revealed. More specifically, the importance of a practical teleplatform should be emphasized.

## Abbreviations

CTCAE: Common Terminology Criteria for Adverse Events
EGFR: epidermal growth factor receptor
QoL: quality of life
